# Research prioritization through prediction of future impact on biomedical science: a position paper on inference-analytics

**DOI:** 10.1186/2047-217X-2-11

**Published:** 2013-08-30

**Authors:** Madhavi K Ganapathiraju, Naoki Orii

**Affiliations:** 1 Department of Biomedical Informatics, School of Medicine, University of Pittsburgh, Pittsburgh, PA 15206, USA; 2 Language Technologies Institute, School of Computer Science, Carnegie Mellon University, Pittsburgh, PA 15213, USA

**Keywords:** Impact prediction, Data analytics, Inference analytics, Protein-protein interaction prediction, Big-data

## Abstract

**Background:**

Advances in biotechnology have created “big-data” situations in molecular and cellular biology. Several sophisticated algorithms have been developed that process big data to generate hundreds of biomedical hypotheses (or predictions). The bottleneck to translating this large number of biological hypotheses is that each of them needs to be studied by experimentation for interpreting its functional significance. Even when the predictions are estimated to be very accurate, from a biologist’s perspective, the choice of which of these predictions is to be studied further is made based on factors like availability of reagents and resources and the possibility of formulating some reasonable hypothesis about its biological relevance. When viewed from a global perspective, say from that of a federal funding agency, ideally the choice of which prediction should be studied would be made based on which of them can make the most translational impact.

**Results:**

We propose that algorithms be developed to identify which of the computationally generated hypotheses have potential for high translational impact; this way, funding agencies and scientific community can invest resources and drive the research based on a global view of biomedical impact without being deterred by local view of feasibility. In short, data-analytic algorithms analyze big-data and generate hypotheses; in contrast, the proposed inference-analytic algorithms analyze these hypotheses and rank them by predicted biological impact. We demonstrate this through the development of an algorithm to predict biomedical impact of protein-protein interactions (PPIs) which is estimated by the number of future publications that cite the paper which originally reported the PPI.

**Conclusions:**

This position paper describes a new computational problem that is relevant in the era of big-data and discusses the challenges that exist in studying this problem, highlighting the need for the scientific community to engage in this line of research. The proposed class of algorithms, namely inference-analytic algorithms, is necessary to ensure that resources are invested in translating those computational outcomes that promise maximum biological impact. Application of this concept to predict biomedical impact of PPIs illustrates not only the concept, but also the challenges in designing these algorithms.

## Background

Big data is everywhere today, be it data from publications (newspapers, journals, internet pages or tweets), or be it cosmological, climatic, or ecological data. This trend is facilitated primarily by the exponential increases in the capabilities of computing, storage and communication technologies. In biology, advances in biotechnology have resulted in the creation of big data of many types: genomic, proteomic, trascriptomic, epigenomic and metabolomic characterizations of several species and their populations.

Large-scale data analytics can aid in discovering patterns in these data to gain new scientific insights; however, the domain of biology is very different from most other domains in this aspect. Validating a computational result is cheap in domains like language translation, and can be carried out by any average individual; when the number of results is in hundreds or thousands, it is possible to crowdsource the validations using Amazon MechanicalTurk [1] or the like. Manual validators can often correct mistakes of the algorithm, and these corrections may in turn be used to improve the algorithm in future. Secondly, the computational results are often ready for direct use or interpretation. For example, outcomes of algorithms for machine translation from one language to another, information retrieval, weather prediction, etc. may be directly deployed for use in real life.

Compared to these domains, there are four fundamental differences in large-scale data analytics for molecular and cellular biology:

(i) Conclusions drawn by algorithms cannot be validated manually, and often require carrying out experiments.

(ii) Even when the computational inferences are accurate and do not require further validation, converting the inferences to meaningful insights requires experimentation.

(iii) Experimental methods require resources that are expensive (material and financial resources) or even unavailable (suitable antibodies).

(iv) Validation and interpretation of inferences requires scientific expertise which may be scarce (for example, there may not be any scientist with expertise in studying some of the proteins).

For these reasons validating or interpreting all the hundreds of computational inferences of data-analytic algorithms is expensive and is not amenable to crowdsourcing.

In this position paper, we introduce the concept called inference-analytics, and a specific inference-analytics problem called impact prediction. We use the terms predictions, computational inferences or hypotheses to refer to the outcomes of data-analytic algorithms.

### Inference analytics for research prioritization

**Figure 1:**
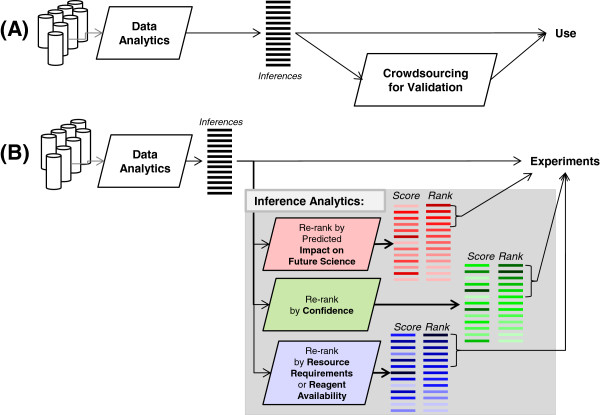
**Inference analytics. (A)** Data analytics typically analyze large datasets to draw inferences; these inferences are usually used directly; the inferences may be evaluated with relatively small investment of resources or through crowdsourcing. **(B)** In areas such as biology, it is desirable that data analytics is followed by inference analytics; these algorithms would analyze the large number of data analytic inferences and re-ranking them by various criteria to aid the users in selecting which inference to pursue. The work presented here corresponds to inference analytics for “scientific impact prediction” criterion.

In domains such as biology where hypothesis verification and interpretation are resource-intensive, the large number of inferences drawn by data-analytic algorithms would have to be reanalyzed by various criteria such as availability of resources (budget, reagents or scientific expertise). We call such algorithms which re-analyze data analytic inferences as inference analytic algorithms (Figure 1); an inference analytic algorithm that is essential in the field of biology is that of predicting the future impact of an inference on biomedicine.

#### Predicting future impact of inferences is necessary in biology and biomedicine

Just as grant proposals are evaluated for their “difference making capability” to determine priority of funding, the inferences drawn at a large scale may be evaluated by their potential for biomedical impact before investing resources to experimentally study them (Figure 1). Impact prediction, or ranking the computational inferences based on future impact is distinct from other methods of re-ranking computational outcomes, such as ranking by confidence of prediction. Here, we assume that all computational outcomes are equally accurate, and propose to re-rank them by their predicted impact on future science. Impact prediction is also distinct from task prioritization, a well-studied area in computer science. Task prioritization assumes that priorities and costs are known for the tasks, and it optimizes allocation of resources to tasks to achieve maximum yield. Here, our goal is to predict the biomedical impact (a type of priority) so that resource allocation (e.g., by a funding agency) can be carried out based on these priorities.

#### Citation count can be used as a surrogate measure for biomedical impact

The focus of this work is on estimating the biological importance of a specific PPI (i.e., how central is a PPI towards understanding other biological or disease related factors). How do we measure this importance? Consider the publications that report each of these PPIs; then consider the publications that cite these original publications. In this narrow domain of publications reporting or citing PPIs, we estimate that each article typically contains one primary result; we measure the importance of a PPI in terms of how many papers cite the original publication reporting that PPI. For example, the PPI of EGFR with Actin was central in advancing our knowledge of several other biological concepts discovered based on this PPI. For this reason, the paper reporting this PPI [2] has been cited by 35 articles in PubMed. The impact of EGFR-Actin is therefore estimated to be at 35 “biomedical results”. Thus, we approximate impact prediction with citation count prediction.

#### Citation count prediction for scientific impact is different from citation count prediction based on metadata

Several researchers have investigated predicting the citation counts for scientific papers [3-5]. However, in these methods only metadata about the publication (number of past articles and past citations of the first author), and content (article title, abstract and MeSH terms) have been used. Our work differs from such work in that we do not consider citation impact based on reputation of the journal or that of the author, but we focus on the biomedical information of the published scientific result that is receiving the citations. Here, citation counts are used as a surrogate measure of the impact of a scientific result. Of course, many factors influence the citing behavior of a given paper: year of its publication, the journal containing the publication, its availability and accessibility, and more [6]. We believe that the original publication would be published in the journal it deserves (e.g., the fact that a paper reporting an interaction was accepted to be published in a high impact journal is in itself an indicator that the interaction may be of high biomedical significance); therefore, the bias introduced by the journal of publication is not an unwanted bias in calculating the biomedical impact. Other factors influencing citation counts, namely the year of publication and years since publication are addressed to some extent in this work. We employ citation counts as indicators of “future impact” of a reported biological result.

### Predicting impact of protein-protein interactions

We demonstrate the idea of inference analytics by developing an algorithm for impact prediction in the domain of PPIs. We propose to predict the impact of each of the PPIs, so that experiments may be prioritized to study the most impactful PPIs.

Although PPIs form the basis of many biological phenomena, 90% of the estimated number of PPIs are currently unknown [7-9]. Extensive research is being carried out both with high-throughput biotechnology and computational methods to discover PPIs [10-14]. Both these approaches provide hundreds or thousands of hypothesized PPIs. The PPI network incorporating these thousands of newly discovered PPIs is useful to directly carry out systems biology studies [15-19]. However, to advance the biology surrounding each PPI (i.e., to translate the inference into biomedical knowledge), many detailed experiments need to be performed (similar to the experiments that reported the importance of EGFR-Actin interaction). These experiments are expensive and time-consuming and most importantly require valuable time of scientists. Selecting a PPI for experimental study is usually based on the feasibility (availability of budget, reagents, time and technical manpower), possibility of formulating a hypothesis of its functional significance, and most importantly, the perceived impact of its validation on advancement of science based on the domain knowledge of the scientist. With this in mind, it is infeasible to study every hypothesized interaction meticulously with detailed experiments due to the sheer number of the inferred interactions.

When viewed from a global perspective, say by a funding agency that has the capacity to drive the science in a direction that is important, the selection of PPIs for experimentation has to be made based on their relative expected impact. There should be a mechanism of identifying what the top 100, say, PPIs are that are most urgent because of their potential impact on biology and biomedicine. Existing algorithms generate thousands of predictions and possibly rank them by confidence, but none currently rank them by the impact that they are predicted to make in the future

## Data description

Binary biophysical PPIs in human were collected from HPRD [20] and BioGRID [21]. HPRD gives PPIs as a list of “binary protein-protein interactions”, and BioGRID gives them marked with the identifier “MI:0407”. These databases present PPIs that are curated from publications, and for each PPI they also give links to the original publication(s) that reported the interaction. The Entrez Programming Utilities [22] was used to retrieve citation information. There were 129,227 references to the 7,581 papers that had one-to-one relationship with interactions. 17,985 of them were self-references (13.92%). We built the interactome network from the PPIs, and computed the centrality measures of PPIs (edges) and the participant proteins (nodes). The resulting network consists of 10,492 nodes and 48,419 edges. A subset of PPIs that have a one-to-one relation with PubMed articles (i.e., those PPIs that are reported by only one publication and where that publication does not report any other PPI) is retrieved to be considered as labeled data for training and test sets.

## Analyses

We propose a computational approach to find the most promising PPIs, and we measure the impact of a PPI in terms of how many papers cite the publication that originally reported the PPI.

Our focus is on estimating the biological importance of a PPI; and we therefore base our predictions on the topological features of the PPIs with respect to the entire interactome. We frame the questions as follows: Is there a detectable trend in the network topology of highly cited interactions or are they placed randomly on the interactome? Do interactions with high biomedical impact have unique, quantifiable network characteristics that distinguish them from other interactions? If we are able to uncover these latent characteristics, naturally, we have strong reason to believe that we should study interactions in the order of their predicted importance. The results show that even by using only the topological features of PPIs, some of the high-impact PPIs can be identified.

Identifying the most important nodes in a large complex network has been well-studied in various fields. In the field of sociology, various centrality measures have been proposed to rank the nodes in a complex network [23]. In the field of biology, these centrality measures are believed to determine characteristics of protein function [24], such as the essentiality of the gene for the organism’s survival [25-28]. In this work, instead of identifying the most important nodes in a network, we consider identifying the most impactful edges in a network.

**Figure 2:**
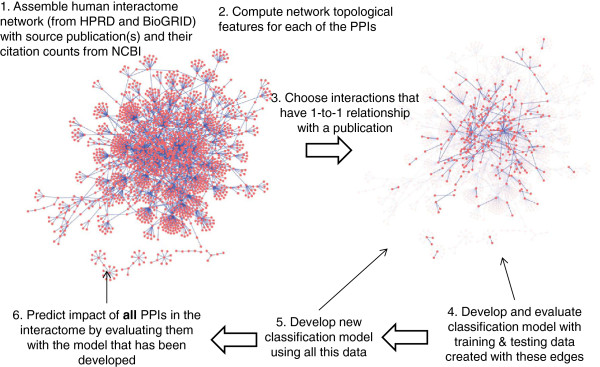
**PPI data.** From the human interactome, those PPIs (edges) are selected that have 1–1 relation with a publication; that is, the publication reports only one interaction, and that interaction is not reported by any other publication. The classification model is trained and evaluated using this 1–1 dataset. After evaluating the approach thus, all of the 1–1 dataset is used to train a new model which is then used to classify each of all of the edges in the interactome to identify high-impact edges. PPI network diagram was created with Cytoscape [29-31].

Figure 2 shows the flow diagram of this work which includes dataset creation, construction of pair-wise features of proteins, development and evaluation of the proposed model, and the application of the model to predict impact of all PPIs in the human interactome.

## Methods

### Feature representation

We computed network centrality measures shown in Table 1 as features, and trained a random forest model to predict high-impact PPIs. Calculation of the node centralities was carried out using the Stanford Network Analysis Library [32], and of the edge centrality using the NetworkX library [33]. As there are 8 node centrality features and 1 edge centrality feature, each interaction is represented by a vector of 17 features, with the first 16 corresponding to the node centralities of the two proteins and the 17th corresponding to the edge centrality. Let *D* = [*X*_1_,*X*_2_,…,*X*_*n*_] represent the *n* training samples and $X_{i}=\left[x_{1}^{\left(i\right)},x_{2}^{\left(i\right)},\ldots ,x_{17}^{\left(i\right)},y_{i}\right]$ represent the *i*-th sample with features *x*^(*i*)^ and class label *y*_*i*_ As PPIs are undirected by nature, and node centralities are features that pertain to nodes (proteins), special care must be taken to treat each interaction as an unordered pair. That is, if we represent an interaction between proteins *a* and *b* by an ordered pair (*a*, *b*), we do not wish to discriminate between (*a*, *b*) and (*b*, *a*). While constructing features *x*^(*i*)^, we let features $x_{1}^{\left(i\right)}$ to $x_{8}^{\left(i\right)}$ be the larger values of the two node centralities, and let features $x_{9}^{\left(i\right)}$ to $x_{16}^{\left(i\right)}$ be the smaller value of the two, in order to symmetrize the feature vectors for (*a*, *b*) and (*b*, *a*).

**Table 1:** Calculated centrality measures

**Feature type**	**Feature**
Node centrality	Degree centrality
	Closeness centrality
	Betweenness centrality [34]
	Eigenvector centrality
	Network constraint
	Clustering coefficient
	PageRank [35]
	Hub centrality (authority centrality)
Edge centrality	Brandes’ betweeness-centrality [36]

### Models

Whether an interaction is labeled positive (high-impact) or negative (non high-impact), is defined by a threshold on the number of citations. For a given threshold *t*, a positive label (*y*_*i*_ = 1) means that the paper reporting the *i*-th PPI received at least *t* citations within the 5-year window following the publication, while a negative label (*y*_*i*_ = 0) denotes that it did not (for details on the choice of the 5-year window, see Results and Discussion). The threshold was set to 5, 10, 30 and 50 in this study. Table 2 lists the number of positive and negative instances under the different threshold settings. Note that we are unable to use interactions reported in papers published less than 5 years prior to this study in the training or test data because we are considering the citation count of a paper within a 5-year window following the publication.

**Table 2:** The number of positive/negative instances and their ratio under the different threshold settings in our dataset

**Threshold**	**Positive**	**Negative**	***n***_***positive ***_***/ n***_***total***_
5	3,393	3,474	0.494
10	1,686	5,181	0.246
30	267	6,880	0.039
50	93	6,774	0.014

Random Forest from the Scikit-Learn machine learning library was used for classification [37-39]. Random Forest has a high prediction accuracy for many types of data achieved by using bagging on samples, random subsets of features, and a majority voting scheme, and has been successfully applied to various problems in computational biology [40]. Random forest also allows the estimation of feature importance [38].

### Evaluation

Prior works in citation prediction have often used the Receiver Operating Characteristic (ROC) [4] or accuracy [5] to evaluate their methods. However, ROC curves can present an overly optimistic view of an algorithm’s performance if there is a large skew in the class distribution [41]. The same holds for accuracy measure. For our task, because there are much more negative instances than positive instances, we use precision and recall, and the area under the precision-recall curve (AUPR) to compare methods. For our prediction task, we are mainly interested in conditions where the false positive rate is low. Thus, we also use R50, a partial AUPR score that measures the area under the precision-recall curve until reaching 50 negative predictions. We perform a 10-fold cross-validation in 20 randomly repeated runs to obtain average values. We repeat cross-validation runs since noise exists in both features and labels; as we shall explain later, our features are calculated from a sampled sub-network and thus inherently contain noise. The averaged performance scores are used for comparison. We compare the random forest model against a random method, in which we assign random probability sampled from a continuous uniform distribution from the interval (0, 1) to each interaction.

### Application to all PPIs in the human interactome

After evaluation of the approach, a new model is trained using all the PPIs with one-to-one relation (i.e., combined training and testing datasets previously used for evaluation). This model is employed to each PPI in the human interactome to predict whether it is likely to have high impact (Figure 2).

## Discussion

**Figure 3:**
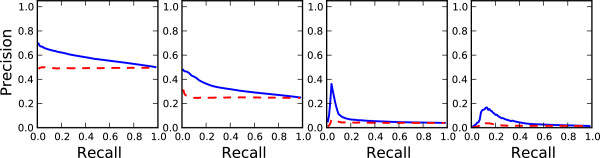
**Precision-recall curve.** Average precision-recall curves are shown for the two methods at thresholds of 5, 10, 30 and 50. The blue solid line corresponds to the random forest model. The red dashed line corresponds to the random probability assignment.

**Figure 4:**
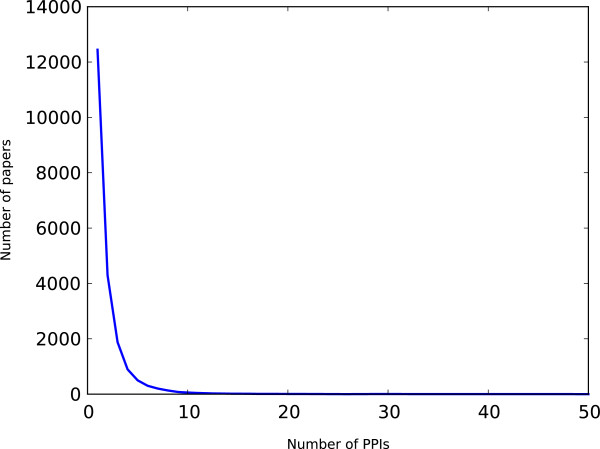
Distribution of the number of PPIs reported in a paper.

Figures 3 and 4 show the distribution of how many PPIs are reported in a paper, and the distribution of how many papers report a PPI, respectively. Most papers report a few interactions, but there are some cases where a single paper reports multiple PPIs. This is due to the fact that HPRD and BioGRID capture all interactions in a paper, even if they are not the main focus of an experiment. We excluded the manuscripts that report a large number of PPIs from training and test data as we do not know which PPIs contributed to the citation counts; the publication may even have been cited for the methodology (such as yeast 2-hybrid or an algorithm). Similarly, we excluded PPIs and corresponding manuscripts where a specific PPI has been reported by multiple manuscripts. This leaves 7,581 interactions among 5,182 unique proteins for training and testing the model. Note that the centrality measures are computed by considering the entire set of PPIs and not just this subset.

**Figure 5:**
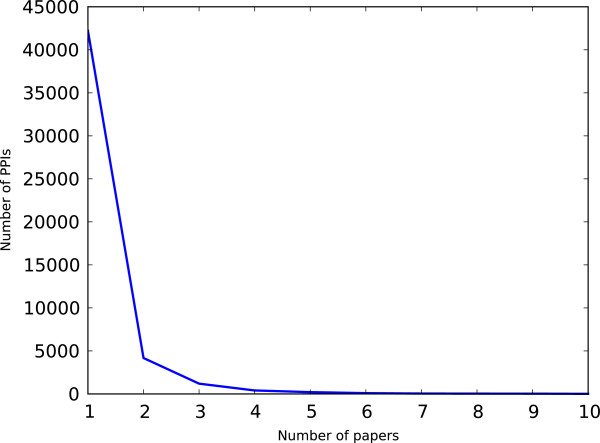
Distribution of the number of papers that report a PPI.

We studied the trends of citation of the papers that report PPIs and investigated whether there is any inherent pattern that underlies the citation behavior for papers that report interactions. Figure 5 shows the temporal citation pattern for the 7,581 papers, separated by the publication year. Regardless of the publication year, most papers tend to have a citation peak within the first 5 years after its publication. Interestingly, there is a second peak after the 5-year window, which corresponds to citations from 2010 to 2012. It is unclear what the reason for this citation peak is. It may be due to the Open Access policy adopted by many journals, authors and funding agencies which started a couple of years prior to 2010, giving the time for other researchers to access the papers and cite them in their work.

Table 3 shows the results of the model in comparison to random baseline. The random forest model with node and edge centrality features consistently outperforms random assignment at all threshold levels with a significance of *P* < 0.01 by the Wilcoxon signed-rank test. Figure 5 compares the average precision-recall curves of the random forest model and random assignment for the different threshold settings.

**Table 3:** Results on the dataset

**Threshold**	**Method**	**AUPR**	**R50**
5	Random forest	0.5718	0.1450
	Random	0.4975	0.0661
10	Random forest	0.3204	0.0762
	Random	0.2510	0.0272
30	Random forest	0.0868	0.0426
	Random	0.0600	0.0197
50	Random forest	0.1115	0.0792
	Random	0.0734	0.0283

Table shows area under the precision recall curve (AUPR) and R50 that measures the area under the precision-recall curve until reaching 50 negative predictions.

**Figure 6:**
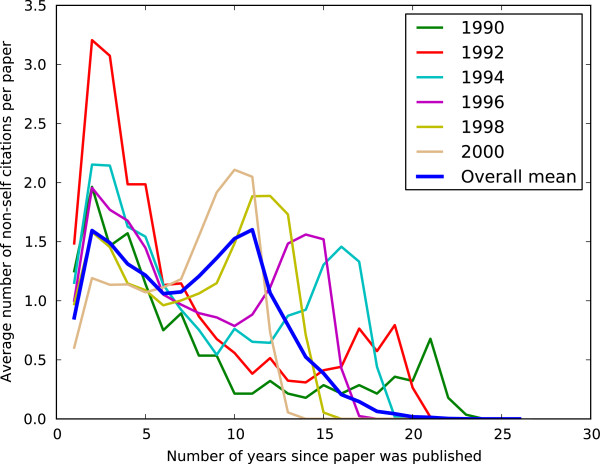
Temporal citation patterns.

**Figure 7:**
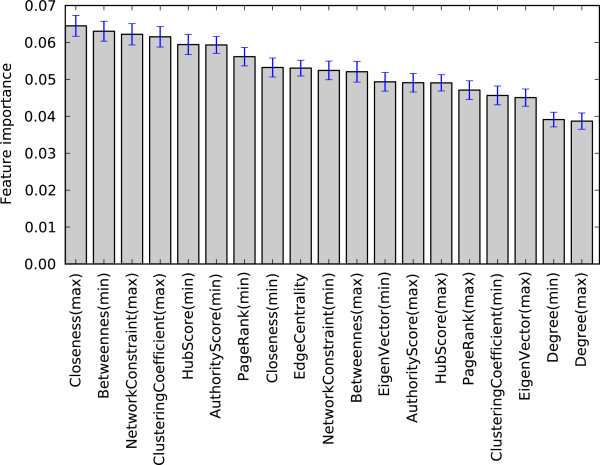
Random Forest Gini importance measures for each feature.

We see that node and edge centrality of an interaction do indeed correlate with how much impact an interaction can make on furthering biomedical science. We assess feature importance based on the Gini index of the random forest classifier. The result is shown in Figures 6 and 7. All of the topology based features are moderately informative, but there are no distinctively dominant features. Prior research has often connected a node’s degree centrality with its essentiality; however, when considering future biomedical impact, we see that degree centrality is the least indicative feature out of all other topology based features.

### High-impact edges in the human interactome

After carrying out the evaluations described earlier, the method was applied to identify high impact edges from amongst all the PPIs in the human interactome. A final model was trained with all available interactions that have a one-to-one relationship with a paper (i.e., including those that were originally left out for evaluation purposes). The model was then applied to identify high-impact PPIs from amongst all the PPIs in the interactome. Note here, that the model was applied on all the PPIs without restricting to those that have one-to-one relationship with publications; such a dataset is required only for training the model accurately and to evaluate the model reliably, whereas, the final prediction of whether a PPI is of high impact is carried out based on its network features alone without dependence on the number of times it has been reported. Table 4 lists the top 10 PPIs that are predicted to be of high impact. As can be seen, most of them indeed resulted in high impact on biomedical science, resulting in up to 413 citations. The top 100 most-impactful edges of human interactome predicted by this model are given in Additional file 1. While these PPIs are predicted to be of high impact, the actual impact achieved by each of these PPIs may be seen on Wiki-Pi web server, which shows up-to-date information on the number of citations received by the publication which reports the PPI [42].

**Table 4:** The top 10 interactions that are predicted to be of high impact

**PPI information**				**Publication information**		
**Rank by impact**	**Gene symbols**	**Protein A name**	**Protein B name**	**Pubmed ID**	**Number of citations so far**	**Year of publication**
1	ADIPOQ - ADIPOR2	adiponectin, C1Q and collagen domain containing	adiponectin receptor 2	12802337	160	2003
2	NMB - NMBR	neuromedin B	neuromedin B receptor	8392057	8	1993
3	NUP93 - TMEM48	nucleoporin 93 k Da	transmembrane protein 48	12928435	37	2006
4	DAO - DAOA	D-amino-acid oxidase	D-amino acid oxidase activator	12364586	72	2002
5	PCM1 - TIC8	pericentriolar material 1	tetratricopeptide repeat domain 8	14520415	99	2003
6	PCM1 - KIAA0368	pericentriolar material 1	KIAA0368	16189514	413	2005
7	BBS4 - PCM1	Bardet-Biedl syndrome 4	pericentriolar material 1	15107855	55	2004
8	SRC - YWHAG	v-src sarcoma (Schmidt-Ruppin A-2) viral oncogene homolog (avian)	tyrosine 3-monooxygenase/tryptophan 5-monooxygenase activation protein, gamma polypeptide	8702721	21	1996
9	GRB2 - SRC	growth factor receptor-bound protein 2	v-src sarcoma (Schmidt-Ruppin A-2) viral oncogene homolog (avian)	11964172	2	2002
10	HCN2 - HCN4	hyperpolarization activated cyclic nucleotide-gated potassium channel 2	hyperpolarization activated cyclic nucleotide-gated potassium channel 4	12928435	26	2003

Note that for each interaction, we only show the publication that has the highest citation count among those that report the said interaction.

#### Functional enrichment of high-impact interactions

We analyzed the statistical enrichment of annotations of the interactions that are predicted to be high-impact. The Gene Ontology term enrichment for proteins involved in the top 50 high-impact interactions has been computed using the BiNGO plugin for Cytoscape [43]. This analysis revealed enrichment in 80 biological processes, 16 molecular functions, and 25 cellular component terms at a statistical significance of *P* < 0.05 (see Additional files 2, 3 and 4 respectively in text format, and Additional files 5, 6 and 7 respectively in Cytoscape format). For example, cell cycle, negative regulation of biological process, and initiation of DNA-dependent transcription are highly enriched biological process terms, while protein-binding transcription factor activity and transcription-factor binding are the highly represented molecular function terms, and nuclear part, membrane-enclosed lumen, and macromolecular complex are the top most significant cellular components.

### High impact interactions of GWAS genes

Genome-wide association studies (GWAS) provide a mapping between genetic factors and diseases by drawing comparisons in the genotype of variants between disease cases and controls. These studies are unbiased by current scientific knowledge about individual genes (i.e., they do not have literature-bias), and identify genome regions with previously unknown biological relevance and provide replicable results [44]. They often uncover several genes of unknown functions possibly participating in hitherto unknown biological pathways [44]. We collected the catalog of GWAS studies which is maintained by National Human Genome Research Institute (NHGRI) [45,46]. This catalog contains 1,309 publications reporting GWAS results on 674 traits or diseases. We investigated whether any high-impact interactions belong to those that are associated with diseases (as identified by GWAS; henceforth referred to as GWAS-genes) [46]. Retinal vascular caliber, type 2 diabetes, and glioma are some of the diseases associated with the genes in the top 100 high-impact interactions. Of the top 50 high-impact interactions, those in which one or both proteins are among the GWAS-genes are shown in Table 5 and a larger list is available in Additional file 8.

**Table 5:** GWAS genes in the top 50 high-impact interactions

	**Publication information**			**Information about genes involved in the PPI**					
**Rank**	**PMID**	**Cite count**	**Pub year**	**Symbol 1**	**Symbol 2**	**Name 1**	**Name 2**	**GWAS diseases/traits of 1**	**GWAS diseases/traits of 2**
1	12802337	160	2003	**ADIPOQ**	ADIPOR2	**adiponectin, C1Q and collagen domain containing**	adiponectin receptor 2	adiponectin levels	
2	8392057	8	1993	NMB	**NMBR**	neuromedin B	**neuromedin B receptor**		Retinal vascular caliber
3	16600873	37	2006	**NUP93**	TMEM48	**nucleoporin 93 kDa**	transmembrane protein 48	HDL cholesterol	
4	12364586	72	2002	DAO	**DAOA**	D-amino-acid oxidase	**D-amino acid oxidase activator**		Bipolar disorder and schizophrenia
8	8702721	21	1996	SRC	**YWHAG**	v-src sarcoma (Schmidt-Ruppin A-2) viral oncogene homolog (avian)	**tyrosine 3-monooxygenase/tryptophan 5-monooxygenase activation protein, gamma polypeptide**		Multiple sclerosis
12	2996780	64	1985	**PRKCA**	SRC	**protein kinase C, alpha**	v-src sarcoma (Schmidt-Ruppin A-2) viral oncogene homolog (avian)	Ventricular conduction,Height	
14	10766163	6	2000	**ESR1**	TP53	**estrogen receptor 1**	tumor protein p53	Bone mineral density (hip),Height,Alcohol dependence,Sudden cardiac arrest,Bone mineral density (spine),Chronic myeloid leukemia,Breast cancer	
15	15173068	8	2004	**ESR1**	GRB2	**estrogen receptor 1**	growth factor receptor-bound protein 2	Bone mineral density (hip),Height,Alcohol dependence,Sudden cardiac arrest,Bone mineral density (spine),Chronic myeloid leukemia,Breast cancer	
16	9568714	278	1998	**GATA4**	**NFATC4**	**GATA binding protein 4**	**nuclear factor of activated T-cells, cytoplasmic, calcineurin-dependent 4**	Retinal vascular caliber	Height
17	10433554	4	1999	**PRKCA**	**YWHAG**	**protein kinase C, alpha**	**tyrosine 3-monooxygenase/tryptophan 5-monooxygenase activation protein, gamma polypeptide**	Ventricular conduction,Height	Multiple sclerosis
18	8266076	105	1993	IL2RG	**IL4R**	interleukin 2 receptor, gamma	**interleukin 4 receptor**		IgE levels
22	12732139	46	2003	**SMAD3**	TP53	**SMAD family member 3**	tumor protein p53	Coronary heart disease,Asthma,Crohn's disease	
23	1454855	45	1992	**PRKCA**	TP53	**protein kinase C, alpha**	tumor protein p53	Ventricular conduction,Height	
24	8266077	115	1993	IL2RG	**IL7R**	interleukin 2 receptor, gamma	**interleukin 7 receptor**		Type 1 diabetes,Ulcerative colitis,Multiple sclerosis,Primary biliary cirrhosis
28	15324660	72	2004	TP53	**YWHAG**	tumor protein p53	**tyrosine 3-monooxygenase/tryptophan 5-monooxygenase activation protein, gamma polypeptide**		Multiple sclerosis
31	15140878	17	2004	**ESR1**	SRC	**estrogen receptor 1**	v-src sarcoma (Schmidt-Ruppin A-2) viral oncogene homolog (avian)	Bone mineral density (hip),Height,Alcohol dependence,Sudden cardiac arrest,Bone mineral density (spine),Chronic myeloid leukemia,Breast cancer	
34	12878187	1	2003	**EGFR**	**PRKCA**	**epidermal growth factor receptor**	**protein kinase C, alpha**	Glioma	Ventricular conduction,Height
36	16192271	88	2005	**ABCA1**	DLG3	**ATP-binding cassette, sub-family A (ABC1), member 1**	discs, large homolog 3 (Drosophila)	MRI atrophy measures,HDL cholesterol,Lipid metabolism phenotypes,Cholesterol,total,Coronary heart disease	
38	17057718	66	2006	**GATA3**	**SATB1**	**GATA binding protein 3**	**SATB homeobox 1**	Hodgkin's lymphoma	Platelet counts
39	15466214	27	2004	**AR**	SRC	**androgen receptor**	v-src sarcoma (Schmidt-Ruppin A-2) viral oncogene homolog (avian)	Prostate cancer,Male-pattern baldness,LDL cholesterol	
44	8994038	82	1997	GRB2	**MAPK1**	growth factor receptor-bound protein 2	**mitogen-activated protein kinase 1**		Multiple sclerosis
46	11782371	15	2002	EP300	**ESR1**	E1A binding protein p300	**estrogen receptor 1**		Bone mineral density (hip),Height,Alcohol dependence,Sudden cardiac arrest,Bone mineral density (spine),Chronic myeloid leukemia,Breast cancer
47	12200137	1	2002	IL2RG	**SHB**	interleukin 2 receptor, gamma	**Src homology 2 domain containing adaptor protein B**		Brain structure
50	7477400	72	1995	HLA-DRB3	**POMC**	major histocompatibility complex, class II, DR beta 3	**proopiomelanocortin**		Type 1 diabetes,Height,Body mass index

Of the top 50 high impact interactions, those in which one or both genes are found to be associated with a disease or trait by genome-wide association studies (GWAS) are shown. GWAS associated genes are shown in bold.

### Open challenges

Inferring biological conclusions from topology of a partially-known network will be influenced by the sampling biases that can alter the underlying structure of the network in unpredictable ways [47]. As mentioned earlier, only 10% of the human interactome is currently known. The fact that we are dealing with a sub-network, rather than a complete network presents an inherent challenge to this study. The first issue concerns the accuracy of the centrality measures: under-studied sub-regions of the network may have superficially low centrality measures, while well-studied sub-regions of the network may have seemingly high centrality measures. The second issue involves bias in the citation behavior: well-studied sub-regions, by definition, are more avidly studied. Thus, it may be the case that well-studied sub-regions will contain highly-cited interactions due to study bias. In order to address this inaccuracy in centrality measures and sociological bias in the citation counts, constructing a network from systematic, unbiased high-throughput experiments (biotechnological or computational methods) is one area of future research.

Impact prediction in any biological domain in general, has unique challenges. Unlike in other computational domains, such as human language translation, it is difficult to estimate the translational impact in biology in a particular study because it is time consuming and requires investment of expensive resources and scientific skills making the design of such algorithms challenging. Despite its difficulties, the proposed direction of research of developing inference-analytic algorithms is necessary to make advances in the most impactful direction.

## Potential implications

High-throughput biotechnology and computational prediction algorithms generate a plethora of hypothesized biological inferences. Meanwhile, high-resolution bench work experiments in biology are often carried out by formulating the hypothesis with a local view of a molecule rather than a systemic view. In this era of scalable data-analytic algorithms which process large data, producing hundreds of inferences, there needs to be a systematic way of determining which of these hundreds of inferences are to be further studied. In this position paper, we propose a new class of algorithms called inference-analytic algorithms that carry out such systematic analysis to prioritize the computational inferences for further study; the concept has been demonstrated in the context of protein-protein interactions (PPIs).

The algorithm is designed to provide a roadmap for the order in which PPIs should be studied further, in the hope that this will prioritize the investment of small-scale experiments and reap maximum benefit for the field of biomedicine as a whole. It has the potential to identify both existing PPIs with untapped impact and also newly predicted PPIs with potential for impact. Factors such as disease-associations and drug-binding also determine which proteins are to be studied further for biomedical impact. On the other hand, factors such as unavailability of reagents may restrict the study of some of these proteins. Future algorithmic prediction of impactful PPIs needs to incorporate these preferences and constraints.

Identifying high-impact PPIs brings the focus of the scientific community onto these proteins and fuels the development of necessary skills, reagents, and so forth.

## Availability of supporting data

Supporting data are made available on BiomedCentral website as described under Additional files section.

## Abbreviations

PPI: Protein-protein interaction; MeSH: Medical subject heading; ROC: Receiver operating characteristics; AUPR: Area under the precision-recall curve; GWAS: Genome-wide association studies.

## Competing interests

The authors declare that they have no competing interests.

## Authors’ contributions

The concept and the design were developed by MKG. The implementation was carried out by NO. The analyses were carried out by MKG and NO. The manuscript is written by MKG with inputs from NO. Both authors read and approved the final manuscript.

## Authors’ information

Madhavi K. Ganapathiraju has Ph.D. from the Language Technologies Institute, Carnegie Mellon University School of Computer Science, and Master’s degree in Electrical Communications Engineering from the Indian Institute Science. She is Faculty in Biomedical Informatics and Intelligent Systems at University of Pittsburgh, where she is applying computational methods to discover hitherto unknown protein-protein interactions in the human interactome. Naoki Orii was graduate student at the Language Technologies Institute, Carnegie Mellon University School of Computer Science when he carried out this work. Subsequent to obtaining his Master’s degree in May 2013, he moved to the data-analytics industry.

## Supplementary Material

Additional file 1 100 most-impactful interactions as predicted by the model, as well as predicted impact scores for all interactions sorted in descending order by predicted impact. Click here for file

Additional file 2 Enriched Gene Ontology terms (biological process) for the top 50 high-impact interactions. Click here for file

Additional file 3 Enriched Gene Ontology terms (molecular function) for the top 50 high-impact interactions. Click here for file

Additional file 4 Enriched Gene Ontology terms (cellular component) for the top 50 high-impact interactions. Click here for file

Additional file 5 Cytoscape session file showing enriched Gene Ontology biological process terms, output by BiNGO. Click here for file

Additional file 6 Cytoscape session file showing enriched Gene Ontology molecular function terms, output by BiNGO. Click here for file

Additional file 7 Cytoscape session file showing enriched Gene Ontology cellular component terms, output by BiNGO. Click here for file

Additional file 8 Of the top 50 high-impact interactions, those in which one or both proteins are among the GWAS-genes are shown. Click here for file
